# Student nurses’ utilisation of self-directed simulation learning at a University in Gauteng

**DOI:** 10.4102/curationis.v47i1.2490

**Published:** 2024-07-25

**Authors:** Lebogang B. Phehla, Agnes Makhene, Lerato Matshaka

**Affiliations:** 1 Department of Nursing, Faculty Health Sciences, University of Johannesburg, Johannesburg, South Africa

**Keywords:** clinical simulation laboratory, self-directed simulation learning, student nurses, time constraints, university

## Abstract

**Background:**

Self-directed simulation learning (SSL) is a globally accepted teaching and learning strategy wherein student nurses take the initiative in diagnosing their learning needs, formulate learning goals, identify resources for learning, and implement relevant strategies in response to their learning needs. This autonomous learning strategy will assist student nurses in taking ownership of their learning. Consequently, student nurses exit the training programme to become lifelong learners, safe and competent professional nurses.

**Objectives:**

This study aimed to explore and describe the experiences of student nurses’ utilisation of SSL at a University in Gauteng and to make recommendation(s) to enhance the use of SSL.

**Method:**

A qualitative, exploratory, descriptive, and contextual research design was used to uncover the student nurses’ experiences with the use of SSL at a University. Nineteen participants were purposively sampled. Data collection was conducted through focus group interviews. Tesch’s method of data analysis was used to analyse, organise and interpret data.

**Results:**

Theme: student nurses experience time constraints, which hinder their utilisation of SSL. Subthemes: (1) a compacted academic timetable, and (2) limited access to the clinical simulation laboratory for self-directed learning.

**Conclusion:**

Time constraints hinder the utilisation of SSL, and this challenge threatens the acquisition of clinical skills and knowledge during the training of student nurses.

**Contribution:**

Evidence-based recommendations to enhance the utilisation of SSL at a University.

## Introduction

According to Forrest, Mckimm and Edgar (2013) simulation is the technique of imitating the behaviour of some situation or process using a suitably analogous situation or apparatus, especially for teaching and learning. The self-directed simulation-based learning (SSL) modality allows limitations of learning in real-life situations to be overcome and can be an effective approach to developing complex clinical skills (Chernikova et al. 2020). In agreement, Cant and Cooper (2017) revealed that SSL has positive effects on student outcomes such as knowledge, decision-making, self-confidence, and self-efficacy. The use of SSL facilitates students in acquiring clinical skills through hands-on training in a safe, zero-risk environment tailored to individual needs (Sahu et al. 2019).

In support, La Cerra et al. (2019) in their study revealed that SSL improves clinical skills and raises confidence and decision-making for undergraduate nursing students. It is worth noting that during the global coronavirus disease 2019 (COVID-19) pandemic, SSL proved to be an essential teaching and learning approach particularly because the teaching and learning of clinical skills could not be swiftly transitioned to online platforms (Dhawan 2020). However, in a study by Reinke (2018), it was revealed that a compacted timetable negatively impacted student learning experiences including SSL. South African Simulation Survey also revealed that simulation-based learning appears to be underutilised because of competing academic demands (Swart, Duys & Hauser 2019).

Therefore, in this study, we explored and described the experiences of student nurses’ utilisation of SSL at a higher education institution (HEI) in South Africa. The study was prompted by marked underutilisation of SSL by student nurses when compared to students from other health science disciplines within the health science faculty. The records of the multidisciplinary clinical simulation laboratory at the host HEI revealed that only 21 out of 168 (12.5%) undergraduate student nurses booked and used the facility for SSL in the year 2019, while 89 out of 111 (80%) undergraduate emergency medical care students booked and used the facility for SSL in the same year. Of the 58 students registered in the Bachelor of Health Science in complementary medicine, 55 (95%) had booked and used the clinical simulation laboratory for SSL. Students do not need to undertake SSL and there are no credits attached to SSL across all the undergraduate professional degree programmes at this host HEI. The year 2020 marked the global outbreak of COVID-19 and access to campuses was significantly reduced in response to the then safety precautions that prohibited gatherings. Therefore, the study aimed to explore and describe the experiences of student nurses’ utilisation of SSL at the host HEI to make recommendations that may enhance the utilisation of SSL among student nurses.

## Research methods and design

A qualitative, exploratory, descriptive, and contextual research design was used to explore and describe experiences of student nurses’ utilisation of SSL at the host HEI (Gray, Grove & Sutherland 2017). The design enabled participants who are directly involved in the utilisation of SSL to bring forth information-rich descriptions on their experiences of utilising SSL at the host HEI (Musarrat, Sudeepta & Ranajee 2019). Furthermore, the design assisted the researcher to mitigate the limitations of time and resources experienced during the study (Bradshaw, Atkinson & Doody 2017).

### Research setting

This study took place in the Faculty of Health Sciences, where the Department of Nursing coexists with 11 other health science disciplines that utilise the clinical simulation laboratory for education and training. The nursing department offers both undergraduate and postgraduate programmes that are regulated by the South African Nursing Council (SANC).

### Population, sample and sampling method

The study population comprised all the 168 (*N* = 168) undergraduate student nurses who utilise SSL at the host HEI. However, the target population in this study comprised 38 (*N* = 38) student nurses in their third year of study as fourth year student nurses were inaccessible because of community nursing science summative clinical examinations that were conducted off campus during the time of data collection. A purposive sampling method was used to draw a sample of 19 (*N* = 19) consenting participants from the target population (Nieswiadomy & Bailey 2018). The participants met the following inclusion criteria:

- Registered in the R425 programme at the host HEI.
- Access to either Microsoft Teams or Zoom meeting platforms and a stable internet connection.
- Willingness to participate in focus group interviews.
- Give a written consent for participation in the study.

This method enabled the researcher to select a sample of experienced student nurses who fits well with the purpose and objective of the study and can bring forth specific, information-rich descriptions about their experiences with the utilisation of SSL (Musarrat et al. 2019). Participants who did not meet the inclusion criteria were excluded from the study.

Invitations to participate in the study and consent forms were sent to the target population by email. Participants who gave informed written consent to participate in the study were sent focus group interview information, and reminder emails were sent a day before the interview session. The participants were made aware of their right to withdraw participation at any stage of the study without consequences.

### Ethical considerations

An application for full ethical approval was made to the University of Johannesburg Faculty of Health Sciences Research Ethics Committee (no. REC-882-2021) and ethical clearance was received on 28 January 2021. The study was underpinned by the ethical principles outlined by Dhai and McQuoid-Mason (2017). These principles include the principle of autonomy, where participants were provided with information about the study. Based on this information, participants individually indicated their willingness to participate by granting written consent. There were no anticipated risks in this study. The principle of beneficence and non-maleficence was achieved by holding interviews virtually to prevent the spread of COVID-19 among the participants, thus ensuring that participants were not exposed to harm during the study. Their wellbeing and interest were safeguarded by conducting interviews at a time that was suitable to their schedules.

### Data collection

Data were collected through focus group interviews facilitated through technologically mediated channels such as Microsoft Teams and Zoom (Basch & Melchers 2021). According to Krueger and Casey (2015), focus group interview is a data collection method where a group of four to six people are assembled to get the participants’ experiences, perceptions, and recommendations on a focused area of interest. The relevance of this method to this study was that the method allowed the researcher to access participants remotely and synchronously while adhering to local COVID-19 safety precautions on social distancing.

After obtaining participants’ contact details from the nursing department, a total of four focus group interviews were conducted, comprising four to six participants each. With the participants’ consent, the 60–90 min long focus group interviews were recorded using a laptop and an external digital audio recorder.

The data-collection process was conducted by an independent facilitator while the researcher adopted an an observer role as the researcher was the primary tool for data collection (Sauro 2015). The independent facilitator was a registered nurse who is trained and experienced in interview skills. The following set of questions guided all the four focus group interviews:

- What are your experiences with the use of the clinical simulation laboratory for SSL at this HEI?
- What are the factors that hinder your utilisation of SSL?
- How can the barriers to SSL be overcome?

The facilitator, researcher, and participants logged in from different locations at the same time. The synchronous interview sessions were individually recorded and transcribed verbatim. Participants were requested to keep their webcams open with their consent to enable the researcher to observe non-verbal cues and any other information relevant to the study. The field notes were recorded on a notepad during each interview session and are included in the data-analysis process. To obtain more clarity and in-depth information from participants, communication skills such as paraphrasing, probing, and reflecting were used (Bryman 2016).

### Data analysis

Table 1 contains a summary of participants’ demographic profile.

**TABLE 1:** Participants’ demographic profile.

Participant no.	Focus Group	Age (years)	Race	Gender	Are you using self-directed simulation-based learning?	Year registered at the selected higher education institution
1.	1	22	Black	Female	Yes	2019
2.	1	23	Black	Female	Yes	2019
3.	1	21	Black	Female	Yes	2019
4.	1	22	Black	Female	Yes	2019
5.	2	31	White	Female	Yes	2019
6.	2	20	Black	Female	Yes	2019
7.	2	22	Black	Female	Yes	2019
8.	2	21	Black	Female	Yes	2019
9.	3	20	Black	Male	Yes	2019
10.	3	23	Black	Female	Yes	2019
11.	3	23	Black	Female	Yes	2019
12.	3	29	Black	Male	Yes	2019
13.	3	21	Black	Female	Yes	2019
14.	3	19	Black	Female	Yes	2019
15.	4	31	White	Female	Yes	2019
16.	4	22	Black	Female	Yes	2019
17.	4	20	Black	Female	Yes	2019
18.	4	22	Black	Female	Yes	2019
19.	4	29	Black	Male	Yes	2019

Data analysis occurred concurrently with data collection wherein the researcher took note of common patterns that were emerging during focus group interviews (Sargeant 2012). Thereafter, the common patterns in the data were identified. Then meaning was linked to the identified patterns to inductively establish themes and subthemes by applying subthemes and themes Tesch’s method of data analysis for qualitative research (Creswell & Creswell 2018).

Furthermore, the researcher read the entire transcript, field notes, carefully listening to audiotape to obtain a sense of the whole (Rutakumwa et al. 2020). The researchers’ thoughts were written in the margin. Similar themes were clustered together and most descriptive wording for the themes were categorised. Ultimately, the theme and subthemes were defined and named (see Table 2).

**TABLE 2:** Emergent theme and subthemes.

Theme	Subthemes
1. Student nurses experience time constraints, which hinder their utilisation of self-directed simulation-based learning	1.1 A compacted academic timetable 1.2 Limited access to the clinical simulation laboratory

In addition, and to strengthen credibility, an independent coder with experience in qualitative research data analysis analysed the data and a consensus was reached between the researcher and independent coder on the theme and subthemes that emerged from the analysis.

### Measures to ensure trustworthiness

Measures to ensure trustworthiness by Lincon and Guba as outlined by Polit and Beck (2017) were used in this study. These measures include credibility, dependability, transferability, confirmability, and authenticity. To achieve credibility, the researcher ensured prolonged engagement by spending more time with participants, building trust between the participants and the researcher, and listening to the audiotape repeatedly. Dependability was achieved by doing member checking, a process whereby the researcher held the fourth focus group interview and asked the same set of questions to ascertain that the themes that emerged and interpretations were representative of student nurses’ experiences with the utilisation of SSL. The provision of thick descriptions of the context within which the study was conducted, participants and research method used was to ensure transferability of the research findings. The rechecking of data during data collection ensured confirmability and that the results are likely to be repeatable by other researchers. Authenticity was ensured by voice recording of interviews and verbatim transcription of the data collected.

## Results

The process of data analysis revealed one main theme: student nurses experience time constraints, which hinder their utilisation of SSL. The subthemes were as follows: (1) a compacted academic timetable, (2) limited access to the clinical simulation laboratory.

### Student nurses experience time constraints, which hinder their utilisation of self-directed simulation-based learning

Time constraints, according to the participants, are largely limitations brought about by unavailability of time to make use of the clinical simulation laboratory for SSL. The student nurses attend classes where theoretical teaching and learning takes place and complete work integrated learning (WIL) at accredited healthcare institutions, thus complying with the provisions of the SANC Regulation No. 425 relating to the approval of and the minimum requirements for education and training of a nurse (general, psychiatric, and community) and midwife to be deemed competent at the end of their training programme. Participants at the host HEI perceived this two-pronged training approach as demanding and quite involved as they must satisfy the SANC statutory requirements by fulfilling the curriculum requirements to proceed to the next level of their training.

When asked on factors that hinder their utilisation of SSL, one participant had this to say:

‘In my view, basically it’s time limitations. We don’t have much time like our weekly schedule is very, very busy … ’ (Participant 11, focus group 3, 23 year old female)

Another participant affirmed and said:

‘Okay, so our schedules [*timetable*] are very congested from first year already. There is time wherein we are expected to be in class for theory and then we’ve got allocated time for simulation laboratory with preceptors, lastly, we need to also cover hours at our allocated WIL sites. The only day that we really have available for self-practice is a Sunday. So, to go into sim lab on the only day that you’ve got off, to try and follow up on practical skills isn’t always possible.’ (Participant 5, focus group 2, 31 year old female)

Some of the participants raised concerns about time constraints citing that time at their disposal is limited and does not permit them to take up SSL. Given the competing priorities in their training programme, participants are often faced with a decision of choosing class attendance and WIL over SSL as SSL is not compulsory and bears no credit to the student nurses at the host HEI.

In order to develop clinical competencies, multiple SSL practice sessions are required to enable a student to competently assess a patient, make a diagnosis, and provide scientifically sound interventions for patient care (Nel & Stellenberg 2015). In agreement with the above literature, Owen (2016) added that practical experience is essential for learning a skill and it may take hours or days to develop competency, and learning all the skills of a craft or a profession may take several years. Therefore, SSL has an essential role to play in the curriculum, to help a nurse gain the necessary knowledge and skills about how to respond to health challenges in clinical areas (Thurling 2017).

It is evident from the participants’ descriptions and the literature that the integration of SSL into the nursing curriculum has become necessary. Such integration has the potential to bridge the time constraints experienced and improve the acquisition of clinical skills. Furthermore, the use of SSL provides an opportunity for student nurses to repeatedly practise and refine clinical skills at their own pace. Consequently, patient health outcome has the potential to improve as adequately skilled and knowledgeable nurses are released into practice. As SSL requires more time than is available, its integration into the curriculum by blocking out time for SSL and attaching credits to the utilisation of this modality by student nurses are recommended to enhance its use among student nurses.

#### A compacted academic timetable

Participants cited that they underutilised SSL, while a few did not have a recollection of the last time they utilised self-directed simulation-based learning. According to the participants, this is because of their compacted academic timetable that does not provide slots for student nurses who have diagnosed their learning needs, formulated their learning goals, to implement SSL in response to their learning needs.

Participants said:

‘Yeah, we are not using the sim lab for self-practice. We are not. I have never heard any of my classmates say let’s book the sim-lab so we can practise a certain procedure, our timetable is heavily loaded. So yeah, we are not utilising it.’ (Participant 6, focus group 2, 20 year old female)

‘Yes. I also agree as in the nursing department I feel like we’re the only department that never makes use of the sim lab for self-directed learning, because our timetable has a lot of things.’ (Participant 4, focus group 1, 22 year old female)

According to Swart et al. (2019), a lack of dedicated time for student nurses to utilise SSL is a barrier towards the uptake of SSL. In addition, a study conducted by Fongang, Atanga and Atanga (2017) revealed that some factors which impede the use of SSL by student nurses were a lack of adequate training on the use of simulation and a compacted timetable. To mitigate these challenges and for SSL to grow as a modality, it needs to be integrated into curricula at the Nursing Education Institution (NEI) level (Swart et al. 2019).

It can be deduced from the above quotes and literature that the formatting of the academic timetable has a bearing on the utilisation of SSL by student nurses. Therefore, the host HEI may need to consider carefully how the integration of SSL into the curriculum is managed to ensure compliance with statutory requirements for education and training accreditation by the regulatory body while enhancing the use of SSL.

#### Limited access to the clinical simulation laboratory for self-directed learning

Participants cited that access to the clinical simulation laboratory was restricted as the clinical simulation laboratory operates from Monday to Friday, between 07:30 and 16:00 at the host HEI. This time schedule denies participants access to utilise the clinical simulation laboratory for SSL during their free time, that is, the time when they are not in class and/or engaged in experiential learning:

‘Around last year we planned it [*self-directed simulation-based learning*] with my friends, we ended up not doing it because we found out that the clinical simulation laboratory closes at 4pm.’ (Participant 17, focus group 4, 20 year old female)

When asked on what can be done to enhance their use of SSL, some participants said:

‘But if the clinical simulation laboratory was operating maybe on weekends [*silence*]. We can actually use that time for self-practice.’ (Participant 12, focus group 3, 29 year old male)

‘… if it’s open [*the clinical simulation laboratory*] from eight till five during the week, that would be fine, we will be able to go and practice.’ (Participant 5, focus group 2, 31 year old female)

‘The operating hours as they stand, makes it very difficult for us to be able to access the sim lab in our own free time.’ (Participant 4, focus group 1, 22 year old female)

Participants displayed willingness to utilise SSL in their free time. However, the clinical simulation laboratory operating hours are restrictive and as such, result in the underutilisation of SSL. A study by Moabi and Mtshali (2022) also recommends that the clinical simulation laboratory operating time be increased to ensure improved utilisation of the simulation laboratories and enhanced students’ learning experiences. According to Aebersold (2018), to ensure improved utilisation of SSL, operating time of the clinical simulation laboratory must be increased. In a study by Carmody, Brown and Del Fabbro (2020), the addition of evening collaborate sessions was another strategy that was very well accepted by student nurses, and students requested more after-hours sessions.

## Discussion

Time constraints, according to the participants, are limitations brought about by unavailability of time to make use of the clinical simulation laboratory for SSL. Participants cited a compacted timetable as one of the reasons they underutilised the clinical simulation laboratory for self-directed learning. Evidence suggests that SSL has a positive role in nursing education and is significantly associated with academic achievement, professional competence, and clinical competencies (Nazarianpirdosti et al. 2021). However, to develop clinical competencies, multiple SSL practice sessions are required to enable a student to competently assess a patient, make a diagnosis, and provide scientifically sound interventions for patient care (Nel & Stellenberg 2015). In agreement with the above literature, Owen (2016) added that practical experience is essential for learning a skill and it may take hours or days [time] to develop competence, and learning all the skills of a craft or a profession may take several years. Therefore, SSL has an essential role to play in the curriculum to help graduate nurses gain the necessary confidence and knowledge on how to respond to health challenges in clinical areas (Thurling 2017).

According to Gatewood (2019), it may be more difficult for student nurses to apply SSL principles when there are competing demands. In agreement, the South African Simulation Survey also revealed that simulation-based learning appears to be generally underutilised (Swart et al. 2019). This is despite the knowledge that SSL opportunities ultimately improve clinical training (Gatewood 2019).

Repetitive deliberate practice with immediate feedback from faculty has been shown to improve learning outcomes (Oermann, Muckler & Morgan 2016), but requires thoughtful planning to schedule and implement. Programme administrators and faculty should consider these factors in deciding how to integrate the use of SSL with other learning experiences across the curriculum (Bradshaw, Hultquist & Hagler 2021).

## Conclusion and recommendations

The study aimed to explore and describe experiences of student nurses’ utilisation of SSL at the host HEI and to make recommendations that may enhance the utilisation of SSL among student nurses. From the findings, it can be deduced that student nurses experience time constraints that hinder their utilisation of SSL at the host HEI. Consequently, student nurses generally underutilise SSL because of competing academic demands. According to the participants, their compacted academic timetable does not provide slots for the use of SSL. Participants recommended that there should be time slots dedicated to SSL on their timetable and others recommended the replacement of some clinical hours with simulation-based learning which includes SSL. Furthermore, the operating hours of the clinical simulation laboratory at the host HEI be extended to 17:00 between Monday and Friday and consideration be made to open the facility over weekends to improve student access and enhance SSL utilisation.

### What this paper adds

The findings of this paper bring to light the fact that student nurses underutilise SSL at the host HEI. Furthermore, it affirms the importance of integrating SSL into the undergraduate nursing curriculum alongside facilitator-led structured simulation-based learning and work-integrated learning to ensure that graduates have clinical competencies and can apply sound scientific clinical judgements when released to practice at the end of the programme. Furthermore, extension of the operating hours of the clinical simulation laboratory helps to accommodate student nurses who wish to make use of the facility for SSL during their free time.
